# Discovery of Marine Natural Products as Promising Antibiotics against *Pseudomonas aeruginosa*

**DOI:** 10.3390/md20030192

**Published:** 2022-03-04

**Authors:** Haoran Li, Mireguli Maimaitiming, Yue Zhou, Huaxuan Li, Pingyuan Wang, Yang Liu, Till F. Schäberle, Zhiqing Liu, Chang-Yun Wang

**Affiliations:** 1 Institute of Evolution & Marine Biodiversity, School of Medicine and Pharmacy, College of Food Science and Engineering, Ocean University of China, Qingdao 266003, China; 17853148949@163.com (H.L.); 17599791586@163.com (M.M.); ouc_zhouyue@163.com (Y.Z.); huaxuan_lee@163.com (H.L.); wangpingyuan@ouc.edu.cn (P.W.); 2 Laboratory for Marine Drugs and Bioproducts, Qingdao National Laboratory for Marine Science and Technology, Qingdao 266237, China; 3 Institute for Insect Biotechnology, Justus-Liebig-University Giessen, Ohlebergsweg 12, 35392 Giessen, Germany; liu.yang@agrar.uni-giessen.de (Y.L.); till.f.schaeberle@agrar.uni-giessen.de (T.F.S.); 4 Branch for Bioresources, Fraunhofer Institute for Molecular Biology and Applied Ecology (IME), 35392 Giessen, Germany; 5 Partner Site Giessen-Marburg-Langen, German Center for Infection Research (DZIF), 35392 Giessen, Germany

**Keywords:** *Pseudomonas aeruginosa*, anti-microbial activity, marine natural product, polyketide, peptide, biofilm

## Abstract

*Pseudomonas aeruginosa*, one of the most intractable Gram-negative bacteria, has become a public health threat due to its outer polysaccharide layer, efflux transporter system, and high level of biofilm formation, all of which contribute to multi-drug resistance. Even though it is a pathogen of the highest concern, the status of the antibiotic development pipeline is unsatisfactory. In this review, we summarize marine natural products (MNPs) isolated from marine plants, animals, and microorganisms which possess unique structures and promising antibiotic activities against *P. aeruginosa*. In the last decade, nearly 80 such MNPs, ranging from polyketides to alkaloids, peptides, and terpenoids, have been discovered. Representative compounds exhibited impressive in vitro anti-*P. aeruginosa* activities with MIC values in the single-digit nanomolar range and in vivo efficacy in infectious mouse models. For some of the compounds, the preliminary structure-activity-relationship (SAR) and anti-bacterial mechanisms of selected compounds were introduced. Compounds that can disrupt biofilm formation or membrane integrity displayed potent inhibition of multi-resistant clinical *P. aeruginosa* isolates and could be considered as lead compounds for future development. Challenges on how to translate hits into useful candidates for clinical development are also proposed and discussed.

## 1. Introduction

**Figure 1:**
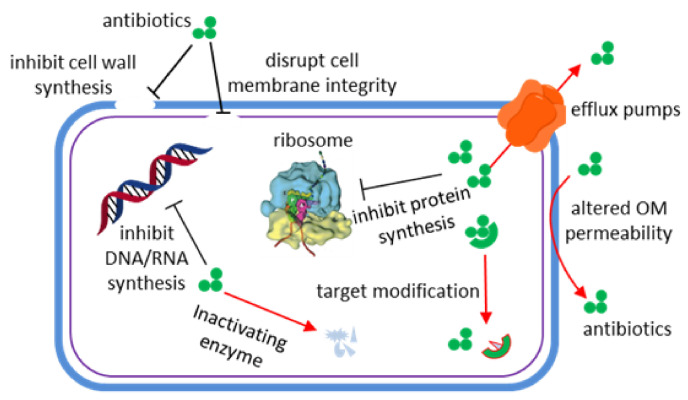
Antimicrobial (black lines) and resistance mechanisms (red arrows) of antibiotics [1].

Infectious diseases have always threatened human health, survival, and development. Antibiotics represent the first solution to combat bacterial infections. Generally speaking, antibiotics take effects through disrupting cell wall synthesis, cell membrane integrity, protein synthesis (e.g., 30S ribosome and 50S ribosome inhibitors), nucleic acid synthesis (e.g., DNA gyrase inhibitors and RNA synthesis inhibitors), or folate synthesis (Figure 1) [1]. However, accompanying their use, drug-resistant bacteria have emerged. Bacteria survive under antibiotic stress via molecular mechanisms including expression of efflux pumps, altered outer membrane (OM) permeability, generation of inactivating enzymes, and target modification [2]. Thereby, the difficulties of curing microbial infectious diseases increased again, compelling people to find new drugs and methods. The common multidrug resistant bacteria in clinic include methicillin resistant *Staphylococcus aureus* (MRSA), vancomycin resistant *Enterococcus* (VRE), carbapenem resistant *Enterobacteriaceae*, and multidrug resistant *Pseudomonas aeruginosa* (MDR-PA) [3]. It should be noted that carbapenem-resistant *P. aeruginosa* was the top category of the “priority pathogens” list published by the World Health Organization (WHO) in February 2017 [4].

As an opportunistic pathogen occurring widely in the environment (as well as the human gut), *P. aeruginosa* causes infections in blood, lung, urinary tract, kidney, and other body parts of immunocompromised patients (especially after surgery) [5,6]. As with other Gram-negative pathogens, *P. aeruginosa* possesses an outer polysaccharide layer, multi-drug efflux transporters, and shows a high level of biofilm formation, together leading to a high probability of antibiotic-resistance [7,8,9]. Even worse, the outer membrane of *P. aeruginosa* is much less permeable (e.g., the porin OprF has a two orders of magnitude lower permeability than OmpF of *E. coli*) and its efflux pumps are more efficient in expelling antibiotics [10]. In addition, gene mutations in *P. aeruginosa* result in enhanced production of antibiotic-inactivating enzymes, overexpression of efflux pumps, and decreased expression of porins [11,12,13,14]. Furthermore, the occurrence of bacterial persister cells give rise to persistent infections and poor prognosis in cystic fibrosis (CF) patients [15]. Thus, *P. aeruginosa* has become a devastating public health threat due to its intrinsic, acquired, and adaptive resistance factors.

Although there are some antibiotics used as monotherapy or combination therapy in clinic to treat *P. aeruginosa* infections, including aminoglycosides (e.g., tobramycin), carbapenems (e.g., imipenem), cephalosporins (e.g., ceftazidime), fluoroquinolones (e.g., ciprofloxacin), Fosfomycin and polymyxins (e.g., colistin), the discovery of new antimicrobial compounds with new modes of action and the potential to overcome resistance is still demanded. In contrast to the rise of multi-drug resistant bacteria, little progress has been made in the introduction of novel antibiotics into the market in the last 20 years [16]. The most recently approved antibacterial drug in 2019, cefiderocol, is a siderophore-conjugated β-lactam antibiotic [17]. There are several anti-*P. aeruginosa* agents in human clinical trials (Table 1), but none of them has made significant progress. The antimicrobial peptide murepavadin failed in phase III clinical trials due to higher than expected acute kidney injuries [18]. Strictly speaking, amitriptyline and QPX7728 are not antibiotics. As an efflux pump inhibitor and β-lactamase inhibitor, respectively, they need to be administrated with other antibiotics. Emerging novel therapeutic strategies in discovery stages includes quorum sensing inhibition, use of iron chelators, and electrochemical scaffolds. However, only a few of them have entered into clinical trials [19,20]. From time to time, new antibiotics against Gram-negative pathogens are discovered, as recently exemplified by darobactin which was discovered from *Photorhabdus* symbionts of entomopathogenic nematode microbiomes and which may share similar requirements for antibiotics with humans [21]. Therefore, there is an urgent need to search for new antibiotics against drug-resistant bacteria to enable human beings to treat infectious diseases.

An investigation on global antibacterial pipeline claimed that over 70% of the projects aiming at new targets are working on antimicrobial peptides (AMPs), natural products, and LpxC inhibitors [22]. Marine natural products (MNPs) derived from marine organisms have proven to be one of the most valuable sources of bioactive small molecules. The marine environment is a special habitat rich in marine animals, plants, and microbes, which can biosynthesize a plethora of structurally unique compounds with significant bioactivities. Among the bioactive MNPs that have been discovered, many demonstrated antibacterial activities with distinctive mode of action. Herein, MNPs isolated in the last decade that are able to directly inhibit the growth or kill *P. aeruginosa* are summarized.

## 2. Emerging MNPs as Promising Antibiotics for Inhibiting *P. aeruginosa*

Marine microbes account for over 98% of the ocean biomass. In order to survive in this biologically competitive environment, marine organisms may produce broad anti-microbial secondary metabolites, which are precious natural resources for the development of antibiotics. It is inevitable to screen the anti-bacterial effects of MNPs. However, inhibition of *P. aeruginosa* is not common. Based on the chemical structures, we have classified these MNPs into anthraquinones, macrolides, alkaloids, peptides, and other structures.

### 2.1. Anthraquinones

**Figure 2:**
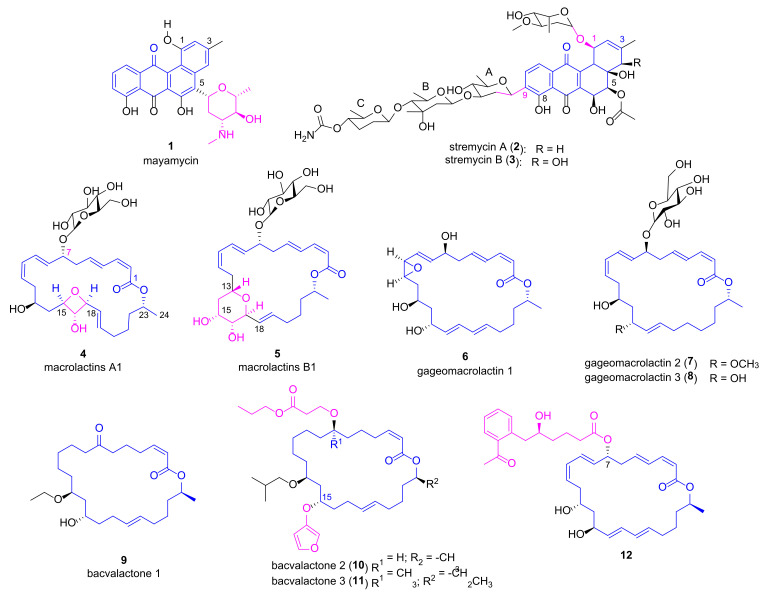
Chemical structures of anthraquinones **1**~**3** and macrolides **4**~**12**.

Mayamycin (**1**, Figure 2), an angucycline-type antibiotic belonging to aromatic polyketide, was identified from a *Streptomyces* sp. strain HB202 isolated from the marine sponge *Halichondria panacea* through variation of the culture conditions [27]. The production of aromatic polyketide mayamycin suggested that HB202 possesses type II polyketide synthases (PKS). Mayamycin glycosylated by a special aminosugar via C-glycosidic bond displayed not only good antibacterial activity against *P. aeruginosa* (DSM 50071) with an IC_50_ value of 2.5 μM, but also nanomolar range cytotoxicity against eight human cancer cell lines. Metal stress strategy (100 μM of nickel) was applied to marine *Streptomyces pratensis* strain NA-ZhouS1 and promoted the production of two new angucycline-type antibiotics with lower prevailing products in traditional culture. It was indicated that nickel stress was able to activate a silencing biosynthetic pathway. The new antibiotics stremycin A (**2**) and stremycin B (**3**) contained 1-position *O*-glycosylation and 9-position C-glycosylation as well as carbamoyl group attached to sugar C [28]. Both compounds exhibited anti-bacterial activities against *P. aeruginosa* with minimum inhibitory concentration (MIC) values of 16 μg/mL.

### 2.2. Macrolides

MNPs macrolactins have been considered as good anti-microbials against both Gram-positive and Gram-negative pathogenic bacteria by inhibiting peptidyl transferase [29]. Glycosylated 24-membered macrolactin A1 (**4**, Figure 2) and macrolactin B1 (**5**) containing oxetane and tetrahydropyran were discovered from two marine bacterial strains *Bacillus* sp. 09ID194 and *Streptomyces* sp. 06CH80 by Shin group [30,31]. Both compounds exhibited inhibitory effects against *P. aeruginosa* with MIC values of 32 μg/mL. Another three 24-membered macrolactin derivatives gageomacrolactins **6**~**8** isolated by the same group displayed much better potency against *P. aeruginosa* with MIC values ranging from 20 nM to 50 nM, whereas no obvious cytotoxic effect was observed on a panel of cancer cell lines (e.g., HCT15 and MDA-MB-231) at the concentration of 30 μM [32]. Bacvalactones 1~3 (**9**~**11**), belonging to 24-membered macrocyclic lactone family, were obtained from *Bacillus amyloliquefaciens* MTCC 12716, a symbiont of intertidal red algae *Hypnea valentiae*. It was proposed that these macrocyclic lactones were generated via a tran-acyltransferase (AT) PKS-assisted *mln* biosynthetic pathway. They showed better anti-*P. aeruginosa* activities (with inhibition zones of 17 mm, 23 mm and 25 mm, respectively) than that of ampicillin (7 mm) [33]. The superior anti-microbial potency of **9**~**10** and **11** compared to ampicillin may attribute to the higher electronic parameter—polarizability (the ability of a molecule to respond to an electric field and acquire an electric dipole moment) [34]. Compound **11** exhibiting the most potent anti-bacterial activities (MIC value of 1.5 μg/mL against *P. aeruginosa* via microdilution method) has the highest polarizability, which may arise from the electron-rich *O*-furanyl, *O*-isobutyl, *O*-propyl propionate, and two additional methyl groups. In order to explore the mechanism of their in vitro bioactivities, molecular docking studies of **9**~**11** with *S. aureus* peptide deformylase (SaPDF) were performed [33]. Aryl-crowned polyketdie (**12**) bearing a 6′-(2″-acetylphenyl)-5’-hydroxyhexanoate group at C-7 position of macrolactin was purified from *B. subtilis* associated with brown seaweed *Anthophycus longifolius* [35]. It displayed comparable anti-bacterial effect against *P. aeruginosa* to positive controls (ampicillin, gentamicin, tetracycline, etc.) with inhibition zone of 15.7 mm and MIC value less than 13 μg/mL. It was proposed that **12** might inhibit pathogenic bacteria through forming a hexadentate Fe^3+^ coordination complex similar to siderophore mode of action [35].

**Figure 3:**
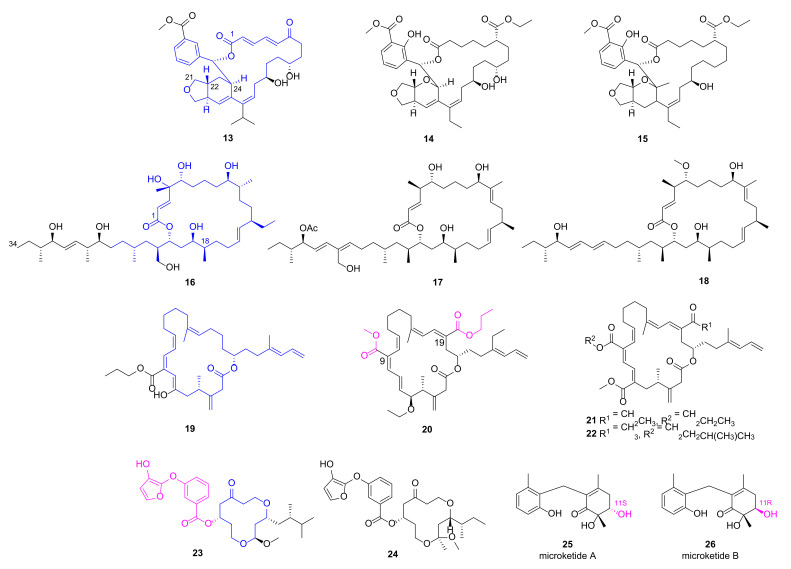
Chemical structures of macrolides **13**~**22** and macrocyclic polyketides **23**~**26**.

Three elansolid-type of polyketide spanned 25-membered macrolides isobenzofuranyl (**13**, Figure 3) and furopyranyl (**14**~**15**), were discovered from marine *B. amyloliquefaciens* MTCC 12716, and anticipated to be biosynthesized through *trans*-AT polyketide synthase of type I PKS. They exhibited impressive anti-microbial activities against *P. aeruginosa* with MICs less than 1.0 μg/mL while the positive antibiotics (ampicillin and chloramphenicol) displayed the MICs more than 6.25 μg/mL [36]. Their in vitro bioactivities were explained through physicochemical parameter and molecular docking studies with SaPDF. Compound 14 has the high topological polar surface area [37] (tPSA, sum of surfaces of polar atoms in a molecule to show its ability to pass membrane) of 158.1, optimal logP value of 3.3, maximum number of hydrogen bond interactions and best drug-likeness score (the odd for a molecule to become a drug which can be predicted by software such as Molsoft^TM^) of 0.98, suggesting it is a promising lead antibiotic. Macrobrevin analogues **16**~**18** encompassing hexahydro-41-hydroxy-macrobrevin-31-acetate functionality were identified from *B. amyloliquefaciens* MTCC12713 via a bioactivity-guided purification strategy [38]. They displayed considerable anti-bacterial activities against *P. aeruginosa* with the inhibition zones of 19 mm, 23 mm and 22 mm, respectively, which is superior than those of positive antibiotics chloramphenicol, and ampicillin (11 mm). Among these polyketide-spanned macrolides, compound **13** had the lowest MIC value (1.56 μg/mL), and its in silico docking study with SaPDF demonstrated a binding energy of 12.61 kcal/mol as well as an inhibition constant (K_i_) of 573.34 pM, implying that it is a promising antibiotic lead compound. Twenty-one membered macrocyclic lactones difficidin analogues **19**~**22**, also isolated from the marine bacterium *B. amyloliquefaciens* MTCC12713, disclosed their significant bactericidal activities with clearance zone of 17 mm, 26 mm, 23 mm and 25 mm, respectively [39]. Among them, compound **20** bearing 9-methyl-19-propyl dicarboxylate demonstrated MIC value of 4 nM against *P. aeruginosa* and drug-likeness score of 0.35. More comprehensive and in-depth studies are needed to validate their activities and investigate the mechanisms in detail. Traditionally, macrolide antibiotics were considered as protein synthesis inhibitors via targeting ribosome, but recent advances have revealed that they probably function as specific translation arrest cofactors [40].

### 2.3. Macrocyclic Polyketide and Microketides

Two new siderophore-type hydroxyfuranyl-benzoate spanned 12-membered macrocyclic polyketides **23** and **24** were isolated from *Shewanella* algae MTCC 12715, and displayed broad antibacterial activities against multiple clinical pathogens including MRSA (inhibition zones of 23 mm and 29 mm, respectively) and *P. aeruginosa* (inhibition zones of 21 mm and 24 mm, respectively) [41]. In order to verify their in vitro anti-microbial activities against MRSA, in silicon docking studies for **23** and **24** with penicillin-binding protein 2a (PBP2a), a transpeptidase that catalyzes cell-wall crosslinking to counter the effect of β-lactam antibiotics. Docking studies indicated that both compounds could occupy the allosteric site of PBP2a with predicted K_i_ values of 17.51 nM and 3.57 nM, respectively. Besides, compound **24** has a higher drug-likeness score of 0.91 than that of **23** [41].

A pair of epimeric polyketides **25** and **26** was discovered by our group from the gorgonian-derived fungus *Microsphaeropsis* sp. RA10-14, and both compounds showed pronounced and broad anti-bacterial activities with MIC values of 0.19 and 1.56 μg/mL, respectively, against *P. aeruginosa* [42]. This result implied that S-configuration at C-11 position is more favorable for the anti-microbial effects.

### 2.4. Alkaloids

**Figure 4:**
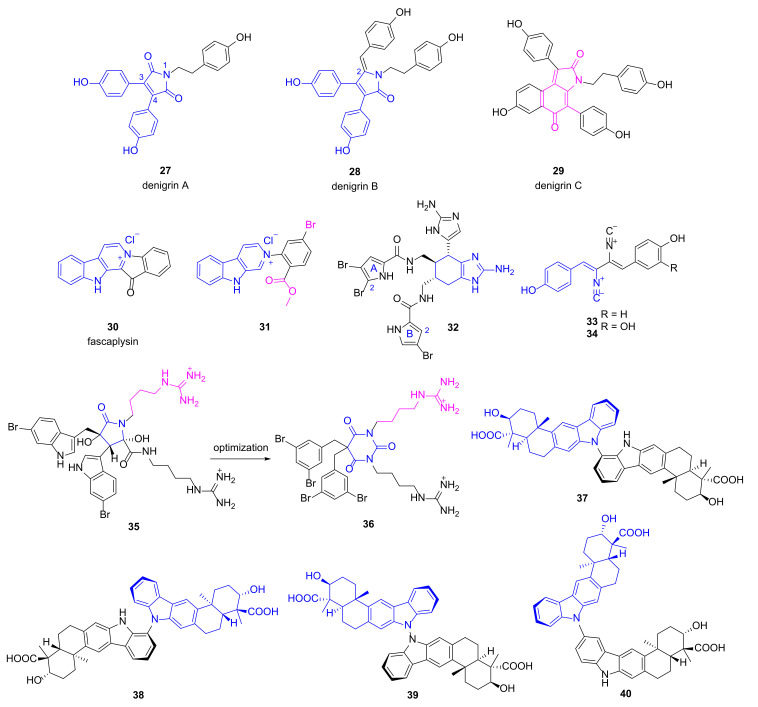
Chemical structures of alkaloids **27**~**40**.

3,4-Diarylpyrrole alkaloids denigrins A~C (**27**~**29**, Figure 4) were isolated from the marine sponge Dendrilla nigra utilizing a bioactivity-guided strategy [43]. Compounds **28** and **29** showed anti-microbial activities against *P. aeruginosa* with MIC values of 25 μg/mL and 12.5 μg/mL, respectively, while compound **27** lacking a p-hydroxyphenyl group at C-2 position just had a MIC value of 100 μg/mL, implying that the p-hydroxyphenyl ring is important for the activity. More efforts are needed to illustrate their mechanism of action. Fascaplysin (**30**), a bis-indole alkaloid with multiple bioactivities, was originally discovered from marine sponge *Fascaplysinopsis bergquist* [44]. Zhidkov et al. designed and synthesized a series of brominated fascaplysins, and compound **31** exhibited potent and selective inhibitory activity toward *P. aeruginosa* with clearance zone more than 35 mm at the concentration of 0.2 mg/disc [45]. It also demonstrated cytotoxic activity against melanoma cells SK-MEL-28 with an IC_50_ value of 1.2 μM.

Jimemez group evaluated the anti-bacterial activities of several known pyrrole-imidazole alkaloids isolated from the sponge *Agelas dilatata* against multidrug resistance (MDR) pathogens including *P. aeruginosa* [46]. Bromoageliferin **32** containing two imidazoles and two pyrroles displayed good and specific inhibitory activity against *P. aeruginosa* ATCC27853 with an IC_50_ value of 8 μg/mL. Moreover, it was effective on four strains of *P. aeruginosa* clinical isolates with a MIC value of 32 μg/mL. Preliminary structure-activity-relationship (SAR) analysis implied that Br at the C-2 position of pyrrole A ring was favorable, while the second Br at pyrrole B ring decreased the anti-bacterial activity. Furthermore, compound **32** was able to inhibit biofilm formation (30~40%) of different pathogens at the concentration of 8 μg/mL and 16 μg/mL. It also increased the survival time (18.3 h vs. 13.5 h) at the dose of 2 mg/kg in an in vivo *Galleria mellonella* model of *P. aeruginosa* ATCC27853 infection [46]. All of these findings make compound **32** a promising lead for the development of novel antibiotics specifically for *P. aeruginosa*.

The known compounds **33**~**34** (Figure 4) possessing diisocyanobuta-1,3-diene were re-isolated by Zhang group from Antarctic-derived *Penicillium chrysogenum* CCTCC M 2020019, and both compounds exhibited broad anti-bacterial activities [47]. Compound **33** has a MIC value of 0.125 μg/mL against *P. aeruginosa*, whereas the appendence of OH group (compound **34**) decreased the anti-microbial activity obviously (MIC value of 8 μg/mL). Meanwhile, both compounds were cytotoxic to cancer cell lines SF-268, MCF-7, HepG-2 and A549 with the IC_50_ values ranging from 0.26 to 5.04 μM [47].

Paulsen and coworkers designed a series of cationic amphipathic barbiturates based on MNP eusynstyelamide (**35**) which is a moderate anti-microbial agent discovered from marine Arctic bryozoan *Tegella cf. spitzbergensis* and the Australian ascidian *Eusynstyela latericius* [48,49,50]. Among the synthesized derivatives, one compound (**36**) with a barbiturate ring replacing dihydroxybutyrolactam ring of MNP **35** showed promising anti-microbial activities against antibiotic susceptible pathogens and 30 multi-resistant clinical isolates including *P. aeruginosa* (MIC values of 4~16 μg/mL) without obvious inhibition on human red blood cells (EC_50_ = 62 μg/mL). It was also efficacious in mice infected with clinical isolates of *Escherichia coli* and *Klebsiella pneumoniae*. Mechanism studies via nuclear magnetic resonance and molecular dynamics simulations suggested that compound **36** took effects through disruption of membrane integrity [48]. Dixiamycins **37**~**40** were obtained from a cold-seep-derived actinomycete, *Streptomyces olivaceus* OUCLQ19-3 by Li group [51]. They all have dual bicyclic substructure fused with carbazole ring connecting via different sites, and exhibited anti-bacterial activities against *P. aeruginosa* with MIC values of 1.56, 1.56, 0.78 and 0.78 μg/mL, respectively [51].

### 2.5. Diphenyl Ethers and Phenols

**Figure 5:**
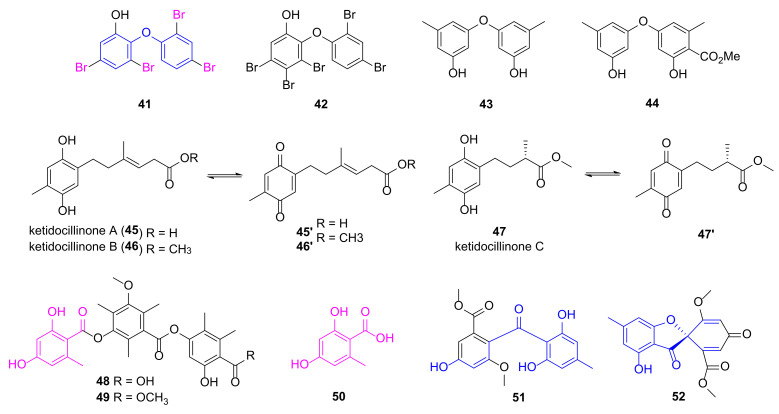
Chemical structures of phenols **41**~**52**.

Polybrominated diphenyl ethers **41** and **42** (Figure 5) were discovered from the marine sponge *Dysidea granulosa* showing in vitro anti-microbial activities [52]. Compound **41** had a MIC value of 16 μg/mL against *P. aeruginosa*, whereas compound **42** with one more Br atom exhibited decreased inhibition against Gram-negative bacteria. The known diphenyl ethers **43** and **44** were re-isolated from marine algae-derived *Aspergillus versicolor* OUCMDZ-2738 cultured with 10 µM of histone deacetylase inhibitor vorinostat in order to regulate the expression of gene clusters which may generate novel MNPs [53]. They specifically inhibited *P. aeruginosa* with MIC values of 17.4 and 13.9 μM, respectively. Phenolic polyketides **45**~**47** were obtained from antarctic sponge-derived fungus *Penicillium* sp. HDN151272 and can be oxidized to quinones **45′**~**47′** easily [54]. Compounds **46** (mixed with **46′**) and **47** (mixed with **47′**) demonstrated potent anti-*P. aeruginosa* activities with MIC values of 1.56 and 6.25 μg/mL, respectively, whereas the MIC value of compound **45** (mixed with **45′**) was more than 50 μg/mL.

Polyketides **48**~**50** sharing a 2,4-dihydroxy-6-methylbenzoic acid fragment were isolated from the mangrove endophytic fungus *Phoma* sp. SYSU-SK-7 by She group displaying potent anti-microbial activities against *P. aeruginosa* with MIC values of 3.27, 1.67 and 2.10 μg/mL, respectively [55]. Compounds **51** and **52**, connecting two phenol groups by carbonyl group and spiro ring, respectively, was isolated by Shao group from soft coral-derived fungus *Aspergillus* sp., and demonstrated good anti-microbial activities against *P. aeruginosa* with MIC values of 7.53 and 3.78 μM, respectively [56].

### 2.6. Peptides

**Figure 6:**
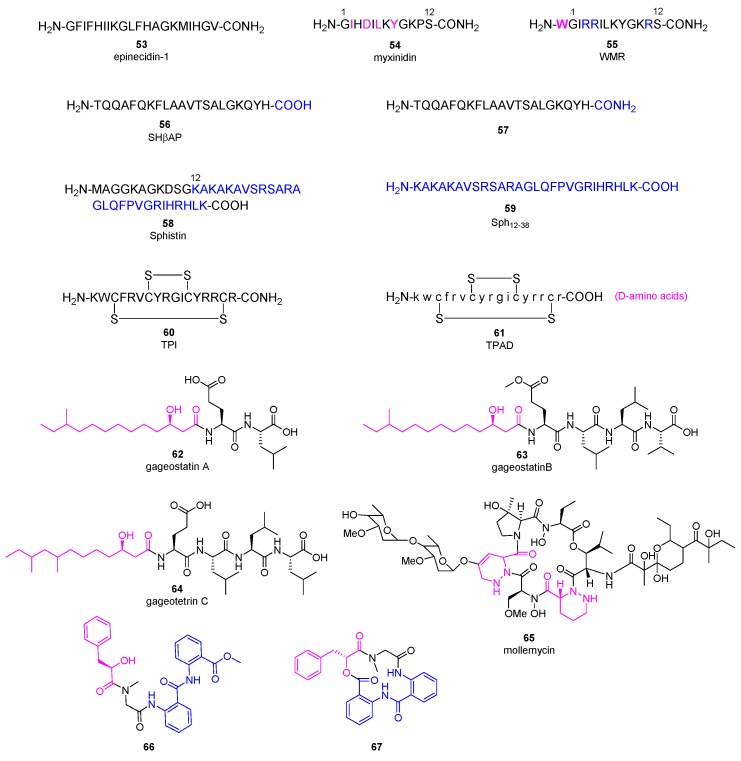
Chemical structures of anti-microbial peptides **53**~**67**.

AMPs as a diverse group of bioactive small peptides were believed to have broad-spectrum activity and less favorable physiochemical properties (e.g., stability and bioavailability). AMPs can kill bacteria alone or synergize with conventional antibiotics. They are able to disrupt membrane integrity, suppress biofilm formation and modulate the host immune responses [57]. New findings and perspectives suggest that AMPs can be remarkably specific, and inferior stability of AMPs means less environmental persistence as well as fewer evolution of antibiotic resistance [58]. Resistance to AMPs results from multiple nonspecific mechanisms such as secretion of proteases and activation of efflux pumps, but AMPs still demonstrate less probability of resistance evolution since they kill bacteria faster and interact with bacterial cell surface rather than directly mutagenic [59,60]. Along with the advances on drug delivery and formulation, AMPs hold great potential combat MDR [61]. Epinecidin-1 (**53**, Figure 6) is a 20-amino-acid peptide produced by orange-spotted grouper *Epinephelus coioides*, playing an important role in protecting fish against Gram-positive and Gram-negative bacteria [62]. Moreover, it was found to be able to inhibit *P. aeruginosa* ATCCS19660 strain and MDR *P. aeruginosa* R strain with the MIC_90_ (the MIC at which 90% of growth was inhibited) values of 50 and 3.12 μg/mL, respectively. Then, its anti-bacterial efficacy in mouse infection models induced by *P. aeruginosa* was evaluated [63]. Compound **53** increased the survival rate of infected mice obviously and decreased the bacterial burden in all explored organs without systemic toxic effects. Besides, it was able to reduce the proinflammatory cytokines at protein and mRNA level. All of the results indicated that compound **53** is a promising lead compound as next generation antibiotic for treating MDR infections.

Myxinidin (**54**, Figure 6) is a 12-amino-acid peptide discovered from acidic epidermal mucus extract of hagfish *Myxine glutinosa* L. displaying anti-*P. aeruginosa* activities with the MBC (minimum bactericidal concentration) values about 7~10 μg/mL [64]. A complete Ala scanning was executed for compound **54**, and the results showed that amino acids at C2, C4, C6, C8, and C12 positions are essential for the anti-bacterial activity [65]. On the contrary, replacement of amino acids at C1, C3, C5, and C11 positions with alanine increased its anti-microbial activities against *P. aeruginosa*. Surprisingly, introduction of tryptophan at the N-terminal and substitution of amino acids at C3, C4, and C11 positions with arginine (peptide **55**) achieved the best potency. Meanwhile, they did not exhibit hemolytic activity at the concentration of 200 μM, suggesting they are promising lead antibiotics with fewer side effects.

Skipjack Hemoglobin β chain-related peptide **56** was obtained from the liver of skipjack tuna *Katsuwonus pelamis*, which demonstrated anti-microbial activity against *P. aeruginosa* with a MIC value of 19 μg/mL without hemolytic activity, even though it cannot cross the bacterial membrane effectively [66]. The C-terminus amidated analogue **57** showed an improved anti-*P. aeruginosa* activity with a MIC value of 5.0 μg/mL.

Sphistin (**58**, Figure 6), a 38-amino acid peptide based on a histone H2A identified from the mud crab *Scylla paramamosain*, exhibited broad anti-microbial activities without obvious cytotoxicity at the concentration of 100 μg/mL [67]. Its truncated fragment Sph12-38 (**59**) gained stronger anti-bacterial effects, and can not only disrupt the membrane integrity but also bind to *Aeromonas sobria* genome DNA at 6 μM [68]. Both the original peptide **58** and its fragment **59** displayed anti-*P. aeruginosa* activities with the MICs of 24 and 12 μM, respectively. Conspicuously, when combined with azithromycin or rifampicin in vitro, both peptides displayed significant synergistic effects against *P. aeruginosa* with fractional inhibitory concentration (FIC, a measure of synergy) index ranging from 0.225 to 0.375 [69]. Surprisingly, mechanism study illustrated that combination of peptide **58** with azithromycin damaged the bacterial cell membrane completely and caused the leakage of cytoplasmic contents while the leakage was not observed in the case of combination of **58** with rifampicin. The in vivo efficacy of peptide **59** in combination with rifampicin or azithromycin was evaluated in a mouse wound model infected by *P. aeruginosa*. Compared to the phosphate-buffered saline (PBS) group, peptide **59** in combination with either rifampicin or azithromycin led to a complete recovery within five to seven or four to five days, while peptide **59** alone or the antibiotic alone could not shorten the healing time.

Tachyplesin I (TPI, **60**) is a 14-amino acid β-hairpin anti-microbial peptide with two disulfide bonds originally isolated from the hepatocytes of *Tachypleus tridentatus* [70]. Replacement of all L-amino acids with D-amino acids in TPI generated TPAD (**61**) which retained the anti-microbial activity (MIC values about 8 μg/mL against *P. aeruginosa*) and showed improved enzymatic stability as well as decreased hemolytic activity [71]. Mechanism study revealed that the activation of the quorum sensing *E. coli* regulators B and C (QseC/B) two-component system induced the bacterial resistance to peptide **61**, while the combination of **61** with QseC/B inhibitor obviously increased the bactericidal effect against several multidrug-resistant bacteria.

Gageotetrins A−C (**62**~**64**, Figure 6), rare linear lipopeptides consisting of a 3-hydroxy fatty acid and di- or tetrapeptide, were isolated from a marine-derived *B. subtilis*, and exhibited good anti-microbial activities against *P. aeruginosa* with MIC values of 0.02-0.06 μM by broth dilution assay [72]. It was speculated that 3-hydroxy fatty acid plays an important role for the anti-bacterial activities of compounds **62**~**64**. Furthermore, none of them are toxic to human cancer cells at 30 μg/mL. The first-in-class glyco-hexadepsipeptide polyketide mollemycin A (**65**) containing two piperazic acids was isolated from a marine-derived *Streptomyces* sp. CMB-M0244, which displayed impressive broad anti-bacterial activities against both Gram-positive and negative bacteria (IC_50_ values of 10~50 nM) [73]. For *P. aeruginosa*, it has an IC_50_ value of 50 nM. Besides, it was able to inhibit drug-resistant malaria parasite *Plasmodium falciparum* with single-digit IC_50_ value without obvious cytotoxicity against mammalian cell line. Aminobenzoic peptides **66** and **67** were obtained from Ascidian-derived endophytic fungus *Aspergillus clavatus* AS-107, demonstrating good anti-bacterial activities against *P. aeruginosa* with MIC values of 32.7 and 8.8 μg/mL, respectively [74].

### 2.7. Pyran Polyketides

**Figure 7:**
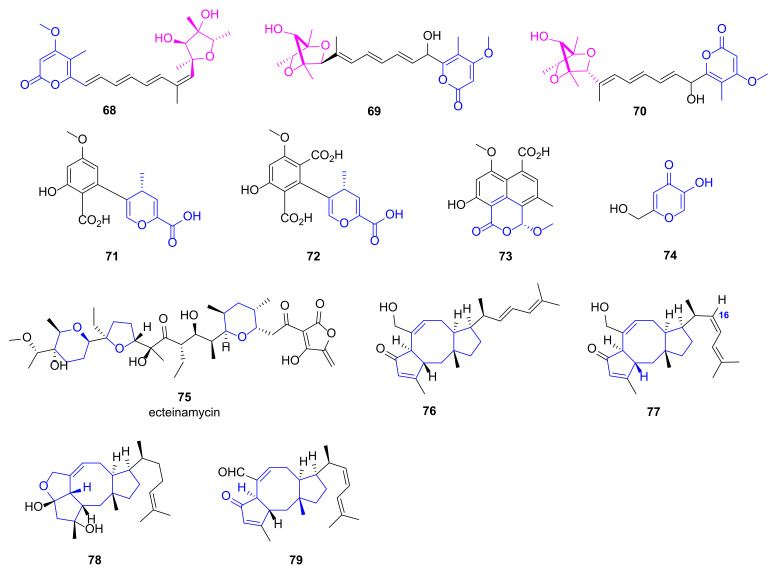
Chemical structures of pyran polyketides **68**~**74**, ecteinamycin **75** and sesterterpenoids **76**~**79**.

Polyene pyrone polyketides **68**~**70** (Figure 7), attaching a furanose or 2,5-dioxabicyclo [2.2.1]heptane pyrone backbone, were obtained from a marine fungus *Penicillium* sp. BB1122, which displayed anti-microbial activity against *P. aeruginosa* with MIC values of 4 μg/mL [75]. It was proposed that they may take effects via inhibiting RNA polymerase and disturbing RNA synthesis similar to myxopyronins. Pyran containing aromatic polyketides **71**~**74** were isolated from a marine *Penicillium* sp. RO-11 displaying potent anti-microbial activities against *P. aeruginosa* with MIC values of 5.2, 1.4, 4.7 and 2.9 μg/mL, respectively [76].

### 2.8. Polyether

Ecteinamycin **75** (Figure 7) was a polyether antibiotic discovered from a marine-derived *Actinomadura* sp. (strain WMMB499), displaying potent anti-bacterial activity against *Clostridium difficile* NAP1/B1/027 in vitro (MIC = 59 ng/mL) and in vivo (mouse model, 30 ng ecteinamycin in 100 μL by oral gavage) [77]. Towards *P. aeruginosa*, compound **75** exhibited inhibited activity with a MIC value of 8.0 μg/mL. It was revealed that compound **75** took effects as an ionophore antibiotic via potassium transport dysregulation based on *E. coli* chemical genomics results.

### 2.9. Terpenoids

Natural products terpenoids have long been discovered with great potential to inhibit microbes via multiple molecular mechanisms involving anti-quorum sensing and membrane disruption [78]. Ophiobolin sesterterpenoids **76**~**79** (Figure 7) were obtained from a deep-sea-derived fungus Aspergillus insuetus SD-512 by Wang group [79]. They all have a polycyclic ophiobolane skeleton and a double-bond-containing side chain. Compounds **77**~**79** demonstrated good anti-microbial activities against multiple pathogens including *P. aeruginosa* (MIC values of 8 μg/mL), *Vibrio alginolyticus* (MIC values of 4 μg/mL) and *Vibrio vulnificus* (MIC values of 8 μg/mL), while compound **76** with a 16-trans configuration did show obvious anti-microbial activity (MIC > 32 μg/mL) [79].

## 3. Conclusions

Oceans are a unique resource for bioactive natural products with great structural diversity and complexity. In a habitat in which microbes constitute >98% of the biomass, many organisms produce antibacterial secondary metabolites to secure survival of the species. MNPs have an inherent high potential to contribute to the discovery and development of novel antibiotics to overcome drug resistance. Their unique chemical structures and explicit anti-bacterial activities probably imply cryptic and novel modes of actions. Thereby, they represent promising treatment of infections caused by Gram-negative bacteria such as *P. aeruginosa*, which has become one of the most threatened pathogens to human health without effective therapies.

**Figure 8:**
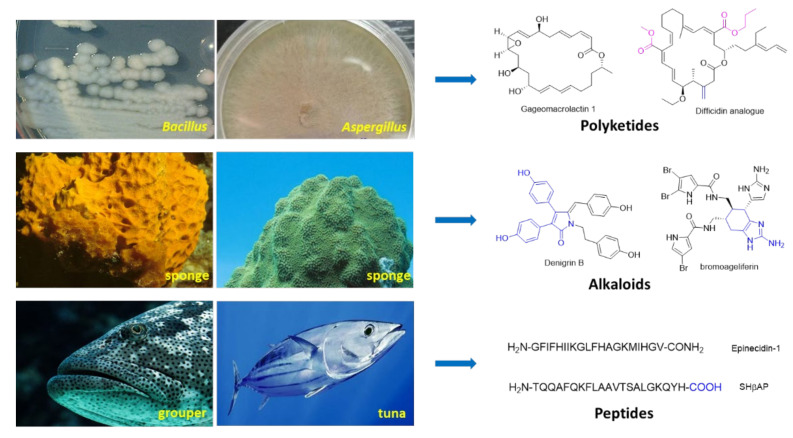
Representative marine organisms producing antibiotics.

In this review, we summarized MNPs discovered in the last decade that possess great potential to be developed as lead compounds for next-generation antibiotics. It is very interesting that for the MNPs we discussed here, most polyketides (e.g., anthraquinone, macrolides, macrocyclic polyketide and pyran polyketides) were discovered from various marine microbes including *Bacillus* sp., *Aspergillus* sp., and *Penicillium* sp., while alkaloids were mainly derived from marine invertebrates (e.g., marine sponge) and peptides were chiefly isolated from marine vertebrates (e.g., fish) (Figure 8). A variety of compounds showed impressive in vitro anti-bacterial activities against *P. aeruginosa* such as MNPs **13**~**15** (MIC < 1 μg/mL), **20** (MIC = 4 nM), **25** (MIC = 0.19 μg/mL) and **39**~**40** (0.78 μg/mL). Several of them (e.g., **32**) displayed good in vivo anti-microbial efficacies in infected animal models. Surprisingly, even though a good amount of MNPs as promising anti-bacterial compounds have been harvested, few novel antibiotics has been launched to the market in the last few decades. Undoubtedly, there are still many obstacles that have to be overcome to develop these hits or lead compounds into clinical drugs. Firstly, most of the MNPs are biologically evaluated preliminary via straightforward assays without further exploration. Thus, secondary assays to confirm their activities are indispensable. Secondly, scaling up of MNPs, which are usually obtained in a limited amount via organic total synthesis or biosynthesis, is needed to provide enough material for more comprehensive investigations on mode of actions and efficacy profiling. Thirdly, nearly all of the approved marine drugs are administrated intravascular, suggesting that the physiochemical properties of MNPs, such as metabolic stability and aqueous solubility, needs to be improved. Even if these MNPs cannot advance into the market, their novel and privileged scaffolds may still be utilized to design anti-bacterial agents. Fourthly, considering *P. aeruginosa*, we have to be aware about the uniqueness of the microbe and the underlying molecular mechanisms of antibiotics, which will enable us to kill *P. aeruginosa* using monotherapy or combination therapy [80]. Additionally, the key factors influencing the transport and accumulation of small molecular antibiotics must be taken into account. It seems that the recent predicted compound accumulation rules do not apply to *P. aeruginosa* [81]. Finally, rather than kill or inhibit *P. aeruginosa* directly, indirect strategies (e.g., *quorum-sensing* inhibitors and anti-biofilm formation) able to enhance the potency of existing antibiotics should be taken into consideration [82,83,84]. In a word, MNPs are anticipated to play important roles and help take a lead in the arms race between bacteria and antibiotics.

## Figures and Tables

**Table 1:** Representative small molecules against *P. aeruginosa* in clinical trials ^a^.

ID	Structure	Phase	MOA ^b^	Indication	Ref.
murepavadin	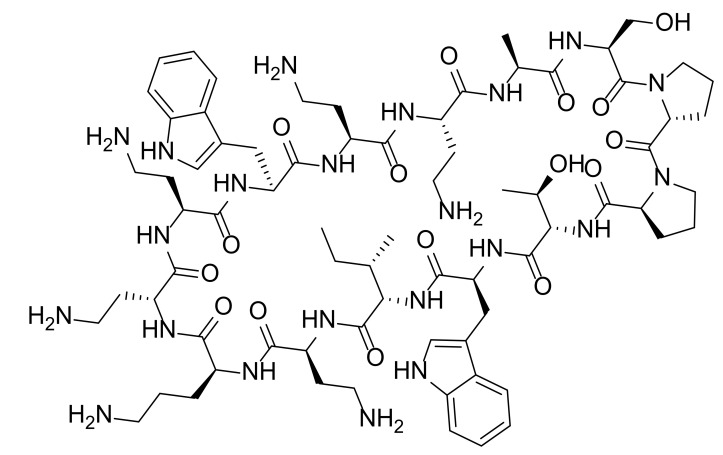	III	LptD inhibitor	Lower respiratory infection; Pneumonia	[23]
fenretinide	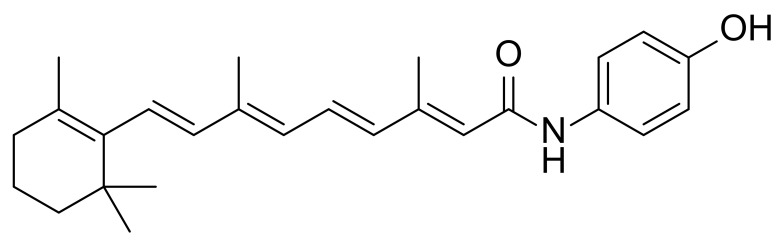	II	--	Cystic fibrosis	[24]
amitriptyline	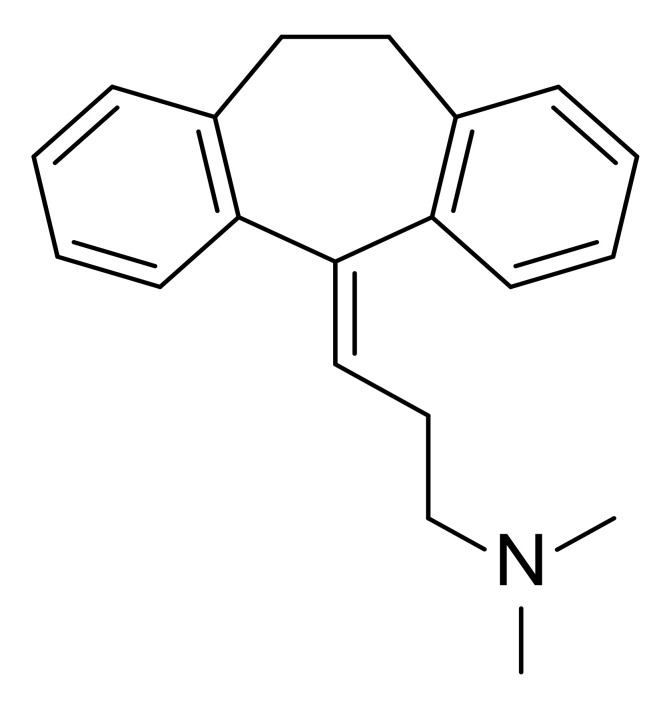	II	Efflux pump inhibitor	Cystic fibrosis; Infection; *P. aeruginosa*	[25]
QPX7728	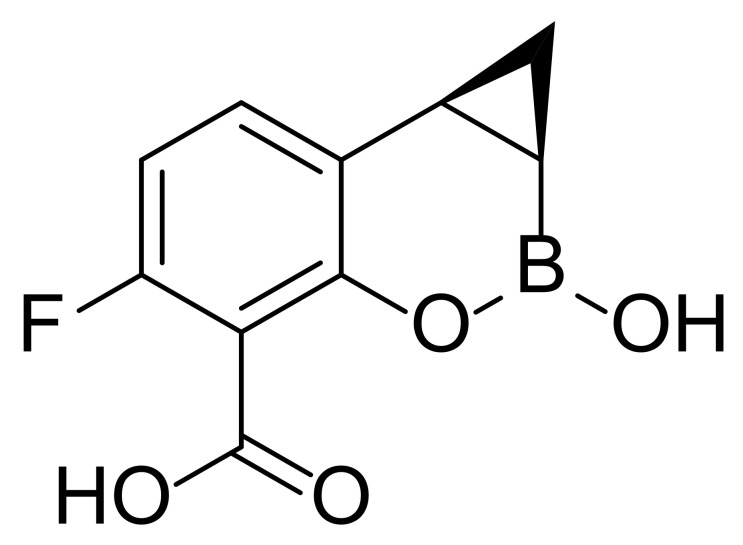	I	*β*-lactamase inhibitor	Bacterial infections	[26]

^a^ Up to date 11 November 2021 from https://clinicaltrials.gov/. ^b^ MOA, mode of action.
